# Simple, robust and near optimal designs for the estimation of log-logistic dose response functions

**DOI:** 10.1007/s00204-026-04339-6

**Published:** 2026-03-10

**Authors:** Tim Holland-Letz, Annette Kopp-Schneider

**Affiliations:** German Cancer Research Center, Im Neuenheimer Feld 280, 69120 Heidelberg, Germany

**Keywords:** Dose-response studies, Optimal experimental design, Robustness, Log-logistic function

## Abstract

Experimental designs in dose response experiments often use simple setups where the dose levels are increased by a fixed factor on the log scale. More efficient or even formally optimal experimental designs exist for this context, but these are often unpopular among applied scientists as they usually depend on the true value of some of the parameters and also frequently propose using only a small number of distinct dose levels. On the other hand, more generally optimal designs such as quasi-bayesian designs are often quite complicated, and still require specifying an a-priori distribution of parameters. In this paper we propose a single graphical representation which shows the performance of any given experimental design under a wide range of possible parameters. Using this representation, we propose four different possible designs which are both simple and still provide reasonable efficiency under many parameter constellations, without needing anything but the most coarse prior knowledge about these parameters. Specifically, our recommended design proposes 10 different dose levels in total, 8 main doses spaced equally around the most likely *ED*50 value, exactly one natural log step apart, and one more set of observations each under control, and under the maximum technically feasible dose. The available observations should be distributed among these dose levels so that each of the main dose levels is assigned roughly $4.7\%$ of the observations, while control and maxdose are assigned roughly $19.6\%$ of the observations, rounding when necessary.

## Introduction

In dose response experiments where the aim is the estimation of a complete dose response function, the selected dose levels for the individual observations have a major effect on the precision of the estimate. In extreme cases, poorly chosen dose levels can even make estimation outright impossible. Optimal design theory for this situation is well developed, and can propose optimal selections of dose levels for nearly any practical situation (Fedorov and Leonov 2013; Holland-Letz and Kopp-Schneider 2015, see also Sebaugh 2011).Using an optimal design means that the desired precision of estimates can be obtained with the minimum number of observations, while inefficient designs might still allow estimation of the parameters, but require more observations to achieve the same precision. Despite this, a recent review (Kappenberg et al. 2023) showed that almost no research papers actually consider or use these optimal designs in practice, instead relying on simple standard designs such as those which place dose levels equidistant on the log scale. The reason for this might be (i) the methodological complexity of the required design theory and the resulting designs which often propose non-intuitive placements and replicate numbers of dose levels, or (ii) the fact that the optimality of designs depends on the true values of the parameters, which cannot be known precisely before the trial, or (iii) existing institutional guidelines or operating procedures which preclude use of certain designs.

In order to improve this situation we propose several designs for log-logistic dose response modeling that try to retain some of the simplicity of the equidistant designs, while still providing reasonably good performance compared to the optimal design, even if any initial parameter estimates deviate substantially from the truth.

To illustrate the performance of these designs in different situations, we introduce the “Robustness Heatmap”, which illustrates the performance of any given design under many different parameter constellations in a single graphic.

The paper is split into four main sections. After the introduction, Sect. 2 will briefly introduce the methodological background of dose response modeling with log-logistic functions, as well as the principles of optimal design for this situation. In Sect. 3 we discuss the effect of prior parameter assumptions on these designs, and introduce the robustness heatmap. In Sect. 4 we then present four different compromise designs which represent different degrees of compromise in regard to efficiency, simplicity and robustness to prior parameter knowledge.

## Background

Dose response experiments generally involve a setup of a (vehicle) control and several increasing dose levels, each applied in several replicates. In many contexts, for example cell culture experiments on well plates, the setup can be controlled precisely, and there is interest in optimizing the experiment in order to best estimate certain dose response parameters, or, more generally, the complete dose response function.

### Functional dose response modeling

Dose response functions are usually modeled by fitting a predefined class of functions to a number of actual observations under different dose levels. A common function used in this context is the four parameter variant of the log-logistic function (notation taken from Ritz 2010)

1 $$\begin{aligned} &f(dose,c,d,b,e50)= c+\frac{d-c}{1+\left( \frac{dose}{e50} \right) ^{b}} \nonumber \\&\quad\quad\quad\quad\quad\quad\quad\quad =c+\frac{d-c}{1+\exp (b(\ln (dose)-\ln ({e50})))}. \end{aligned}$$

The parameters *c* and *d* represent parameters determining the lower and upper limit of the dose response curve, *b* determines the slope (also known as the “Hill slope”) and *e*50 (sometimes called just *e*) represents the position of the dose level where $50\%$ of the maximum relative effect can be observed (*ED*50). In this context, positive values of *b* indicate decreasing functions, while negative values describe increasing functions. The parameters *c* and *d* are scale parameters for the response only, otherwise not affecting the general shape of the curve. Values of *c* and *d* of 0 and 1 result in responses between 0 and 1, and are considered the “standard” parametrization, often paired with values of 1 for both *b* and *e*50 as well. Effects of changes in the parameters are illustrated in the appendix. Note that in case of increasing functions (i.e. negative values of *b*) the scale parameters *c* and *d* switch their interpretation, but this does not affect the results in this paper.

In practice, the actual parameters *c*, *d*, *b* and *e*50 can be estimated from a data set of observations under increasing dose levels using nonlinear least squares or maximum likelihood estimation.

For the more general case, we consider the dose response function *f* to be a function $f:\mathcal{X}\times \varTheta \rightarrow \mathbb {R}$ based on a design space $\mathcal{X}$ consisting of *l* different available dose levels and a parameter vector ${\boldsymbol{\theta }}=(\theta _{1},...,\theta _{k}) \in \varTheta$ taken from a parameter space $\varTheta \subset \mathbb {R}^k$. We assume *f* to be differentiable in $\boldsymbol{\theta }$.

For the log-logistic function we thus have $\boldsymbol{\theta }=(c,d,b,e50)$ and $\mathcal X$ as the set of all dose levels which could theoretically be used in the experiment, including the control. Often, non-control dose levels are understood as decreasing dilutions of the substance, and available dose levels can thus be described as a set of increasing dose levels equally spaced on the log-axis. For our examples, we will always consider a design space consisting of log-dose levels between $-10$ and 10 increasing in increments of 0.1 natural log steps and centered around a natural log dose of 0, all measured in suitable units. The log dose level of $-10$ is used to indicate measurements under zero dose in practice, i.e. the control. Deviations caused by this approximation are completely negligible. Thus, $\mathcal{X}$ is defined as $\mathcal{X}=(\exp (-10),\exp (-9.9),...,\exp (10))$ and will contain $l=201$ possible dose settings. Please note that all logarithms used in this paper refer to natural logarithms at base *e*, denoted as $\ln ()$.

### Basics of optimal experimental design

Estimating the unknown parameters for a specific substance requires multiple observations at different dose levels, and the precision of the estimates depends on the sample size as well as on the specific dose levels used in the experiment (Fedorov and Leonov 2013). Any specific selection of dose levels for the experiment is called the design of the experiment (usually denoted with $\xi$). Such a design $\xi$ will always consist of a vector identifying a number of different dose levels to be used, and a second vector of the same length assigning specific weights (values between 0 and 1) to each dose level. If for example a design proposes the dose level x with a weight of 0.25, this means that $25\%$ of the available observations should be taken at the dose level x as replicates. In raw numbers this means that if $n=100$ observations are available, 25 of these should thus be taken at a dose level of x. That way, any design $\xi$ can be expanded into an actual set of *n* different observations $(x_1,...,x_n)$ for the experiment, distributed among $< n$ distinct dose levels, with replicate numbers chosen according to the design. Weights can be different for each dose level, i.e. a design will not necessarily use the same number of observations for each dose level. In total, however, all weights in a design will always sum to 1.00, i.e., $100\%$ of observations.

The design giving the best precision of the parameter estimates (in some sense) is called the optimal design $\xi _{opt}$ for this situation. For this, optimality of a design $\xi$ is judged based on the inverse of the variance matrix for the parameter estimates to be expected when using this design. This matrix is called the Information matrix $M(\xi , \boldsymbol{\theta })$ of a design $\xi$, and it is constructed from the derivatives of the dose response function in direction of the parameters of interest (see Appendix for more details). The larger the elements of this matrix are, the more information is available about the corresponding parameter.

As as single design cannot be optimal for the estimation of every single parameter at once, designs generally try to achieve either a balanced optimization of all parameter estimates (*D*-optimality, expressed through the determinant of the information matrix $|M(\xi , \boldsymbol{\theta })|$), or focus on specific linear combinations of the parameters (*c*-optimality), see (Silvey 1980) for both. We will focus on *D*-optimality here.

Optimal designs for concrete situations can usually be found using algorithmic approaches. We use the general algorithm proven by Yu (2010), but many other, more sophisticated variants exist (e.g. Yang et al. 2013).

### Dependency of optimal designs on parameter assumptions

Unfortunately, the exact dose levels needed for an optimal design generally depend on some of the true parameters of the model (“locally optimal designs” (Chernoff 1953), designs are only optimal for estimating parameters close, that is, “local”, to the parameter settings used to derive the design). In case of the log-logistic function these are specifically the parameters for the slope (*b*) and for the *ED*50 level (*e*50), while the scale parameters *c* and *d* do not affect the designs in any way at all. As the parameters *b* and *e*50 are by design not well known before a dose response experiment, there will be some practical ambiguity in regard to the truly optimal design in any given situation. In practice, prior guesses will be made based on either a small sample size range finder trial, or based on results from earlier phases, literature results or the properties of similar substances. In this paper, we will propose some designs which should perform decently even when the prior information is somewhat unreliable.

## Assumption-robust designs

As the optimality of a design depends on the true values of the parameters, it is of interest to assume certain parameters $\boldsymbol{\theta }$ and then compare the performance of any given design $\xi$ to the performance of the actual optimal design that was derived for exactly these parameters ($\xi _{opt}$). This is called the (local) efficiency of a design under a given parameter vector $\boldsymbol{\theta }$:

$$\text {Eff}(\xi ,\boldsymbol{\theta })=\root k \of {\frac{|M(\xi , \boldsymbol{\theta })|}{|M(\xi _{opt},\boldsymbol{\theta })|}}$$

for the *D*-optimality criterion, with *k* being the number of parameters in the model, i.e. $k=4$ for the log-logistic function (1). Note that efficiency is proportional to the required sample size, e.g. a design with $50\%$ efficiency requires twice as many observations to achieve the same performance as a design with $100\%$ efficiency.

Any design $\xi$ which has an efficiency close to 1 for a wide range of different true values of $\boldsymbol{\theta }$ can be called a *robust* design.

### Transferability of optimal designs

**Fig. 1:**
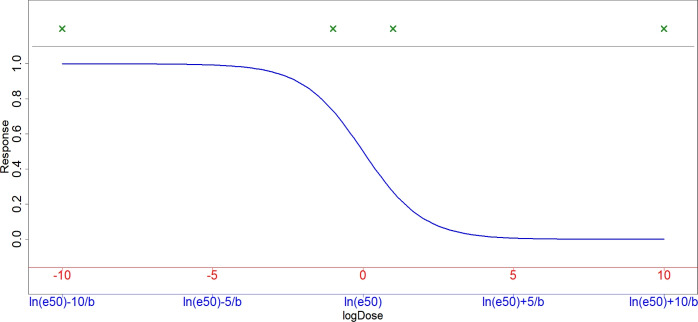
Log-logistic function under standard parametrization (red axis) and under generic parametrization (blue axis). Green marks at the top show doses for the locally optimal design under either parametrization (each used for $25\%$ of observations)

Unfortunately, calculating the efficiency of a design under different parameters requires knowledge of the respective optimal designs under each of these parameter sets. Even with efficient algorithms, this requires considerable computational effort. Fortunately, however, at least for four-parameter log-logistic functions, it is possible to directly and analytically calculate optimal designs for any parameter constellation from a single known optimal design. We will show this property as a special case of a more general relationship. The resulting Proposition is somewhat technical and can be found in the Appendix. However, applied to the log-logistic function it states that when an optimal design for the standard parametrization (${\boldsymbol{\theta }} = (c,d,b,e50) = (0,1,1,1)$) is known, the optimal design under different parameters can be generated by dividing each of the old log dose levels by the new value of *b* and then shifting the resulting values by the new $\ln (e50)$. Here, “old” dose levels refers to the doses proposed by the optimal design under the standard parametrization, while “new” dose levels refers to the updated dose levels required for an optimal design assuming the changed parameter values. Heuristically, the reasoning behind the Proposition can be seen from Fig. 1. The figure shows the log-logistic dose response function as well as the locally *D*-optimal design. The x-axis in red shows the standard parametrization, while the blue axis indicates the general formulation. As the function retains the same shape and the only difference is a shifting and stretching of the x-axis, any existing design for the standard formulation can be equivalently shifted to obtain a design of the same efficiency for a different set of parameters *b* and *e*50.

As an example, assume a 4-dose design for the standard parametrization ($b=1$, $e50=1$) has been derived, proposing $25\%$ of observations each to be taken at log doses of $-10,-1,1,$ and $+10$. Now, lets say new information has been obtained and the new best guesses for the parameters are $b=2$ and $e50=3$. The previous design can now be shifted by first dividing each log dose level by 2, and then adding $ln(e50)=ln(3)$ to each of the resulting values. The resulting design will thus propose log dose levels of $(-5+ln(3)), (-0.5+ln(3)), (0.5+ln(3))$ and $(5+ln(3))$, still to be used at the same weights of $25\%$ each. This design will perform equivalently to the previous design in case the new parameter assumptions are the correct ones.

In regard to more general dose response functions different from the log-logistic model, this property still holds whenever, after an analogous design shift, the derivatives of the dose response function can be cleanly split into separate parts which depend either (i) only on the parameters or (ii) only on the dose levels, but in no part depend on both simultaneously. See the Appendix for more details.

### The robustness heatmap

We propose to apply the Proposition to log-logistic functions to calculate the efficiency of any given experimental design for a wide grid of possible true values of *b* and *e*50, in both cases expressed as equally spaced values on the log-scale. For *b* we consider a grid of *b*-values increasing by a factor of 20 from smallest to largest values, with $b=1$ as the midpoint. On the log-scale, this corresponds to potential misspecifications by up to 1.5 natural log steps (factor of 4.48 in raw doses) in either direction. For *e*50 we consider a grid of possible values corresponding to a dilution factor of 400 between the smallest and largest *e*50 value, centered around an assumed value of $e50=1$ as the midpoint (equivalent to deviations of the true *e*50 by up to 3 natural log steps in either direction, i.e. a factor of 20 in either direction). In our experience, this range covers most reasonable misspecifications for expected values of *b* and *e*50 for different substances, but this is of course highly context dependent. For example, in the cell culture based ACuteTox project (Clothier et al. 2013), we found values of *b* for a wide variety of substances tested on 3*T*3 cell lines in different labs in different European countries to differ in magnitudes by no more than 3–4 log steps. However, possible *e*50 values of different substances varied by many magnitudes, and a universal design is thus not possible in regard to this parameter. Thus, any design presented here still requires a rough prior guess of the $\log$-*e*50-value, and this guess should be used as the midpoint of all designs proposed, instead of the default dose of 1 unit (log dose of 0) assumed here. Based on this setup, robustness of designs will then be assessed for deviations up to 3 log steps in either direction from this assumption. Of course, prior knowledge of *b* can and should be used in the same manner, but having prior information is less crucial for this parameter.

**Fig. 2:**
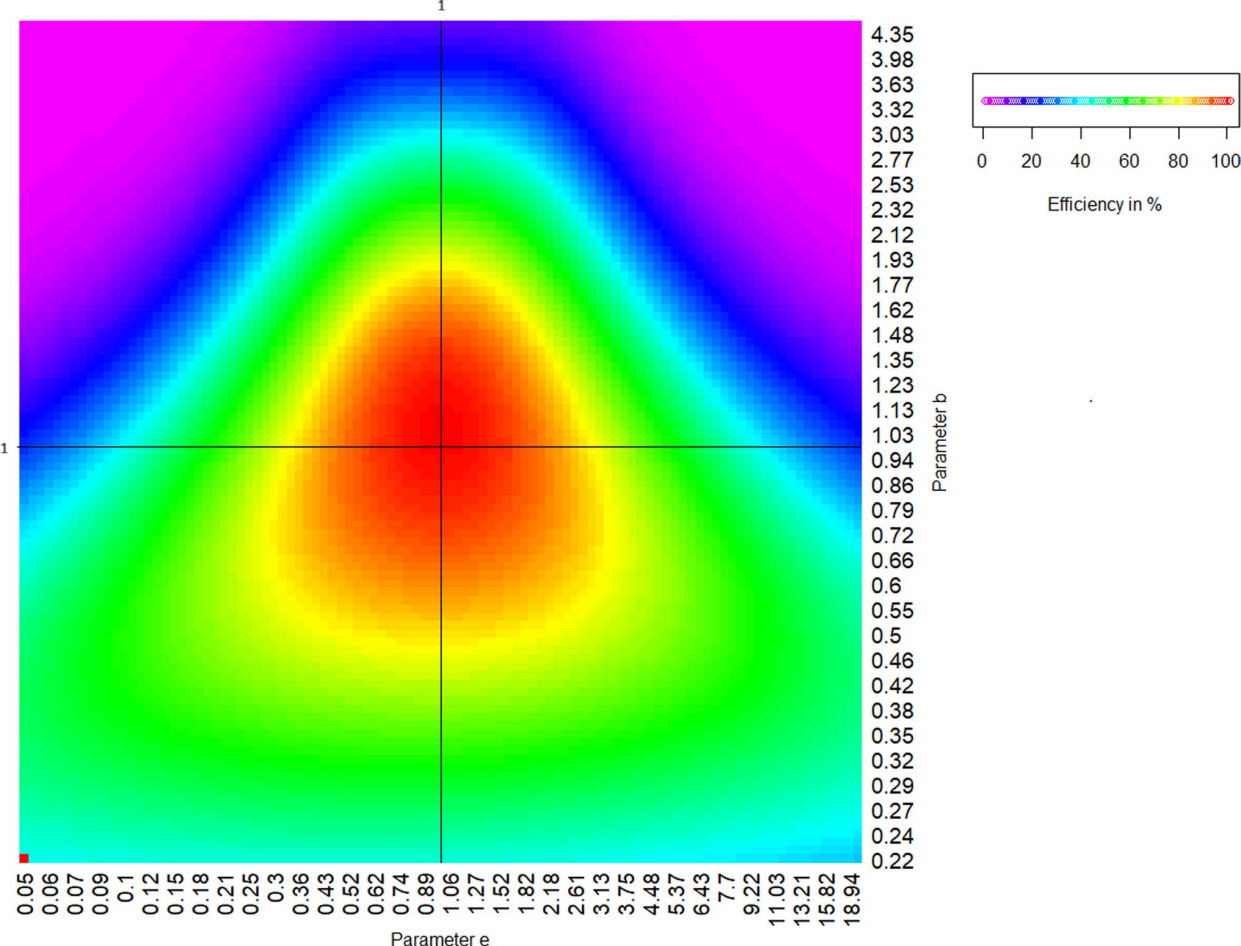
Robustness heatmap for the optimal design under standard parametrization ($\theta =(0,1,1,1)$). Colors indicate efficiency under different combinations of true parameters *b* and *e*. Note that the red pixel on the lower left has been placed there purely for color calibration reasons and does not represent a real result

Next, we divide the parameter ranges for both parameters selected above into a grid of 101 natural log steps each, resulting in a total of 10201 parameter combinations, requiring the same number of efficiencies to be calculated. We plot these efficiencies in a two-dimensional heatmap, with values for *e*50 and *b* on the x- and y-axes, respectively, while the corresponding efficiencies of the proposed design at each parameter combination is indicated through colors. For the 4-point optimal design proposed in Holland-Letz and Kopp-Schneider (2015) and shown in Fig. 1, the resulting heatmap is shown in Fig. 2. While this design is optimal only in case the exact parameter assumption used in the construction holds true (${\boldsymbol{\theta }} = (0,1,1,1)$, called a “locally optimal design” for this parameter set), we observe that it still performs reasonably well for small and moderate deviations from the assumptions.

To be more specific, if we observe the heatmap we see that the design shows a red color at the $(b,e50)=(1,1)$ parameter intersection, showing us that the design indeed has $100\%$ efficiency under the parameter settings it was derived from. However, if the true parameters are different, the efficiency generally will be less. For example, if we assume the true parameters are $(b,e50)=(0.72,3.13)$, i.e. a moderate deviation, the heatmap shows us a yellow color, indicating that the previously optimal design now has an efficiency of only about $80\%$. The design is still workable, but switching to a different design can save up to $20\%$ of observations. However, greater deviations, especially if both parameters change, quickly result in substantial loss of efficiency. For example, under $(b,e50)=(1.62,6.43)$, the heatmap already shows a deep blue color, indicating less than $30\%$ efficiency.

In addition to being an intuitive visualization, the underlying calculations of the robustness heatmap also allow several overarching performance metrics:

- The performance at the a-priori assumed parameter values $\boldsymbol{\theta }_{prior}$ (the local efficiency $\text {Eff}(\xi ,\boldsymbol{\theta }_{prior})$).
- The average performance over the whole parameter range (i.e. the average efficiency of all robustness heatmap entries, expressed mathematically as the integral of the efficiencies over all possible true parameter values in the heatmap using equal weights)
- A weighted average over the parameter range, based on an a-priori probability that these parameters are true (Bayesian average efficiency, see Sect. 4.2, expressed mathematically as the integral of the efficiencies over all possible true parameter values in the heatmap using custom weights)

A formal mathematical representation of all three metrics can be found in the Appendix.

Other criteria are possible, such as the performance at any specific constellation or even at the best parameter combination for this design (i.e. the maximum over the robustness heatmap). We will however focus on the three listed criteria here.

In the next main section, we will use these measures to present and compare several experimental designs.

### R-Shiny implementation

**Fig. 3:**
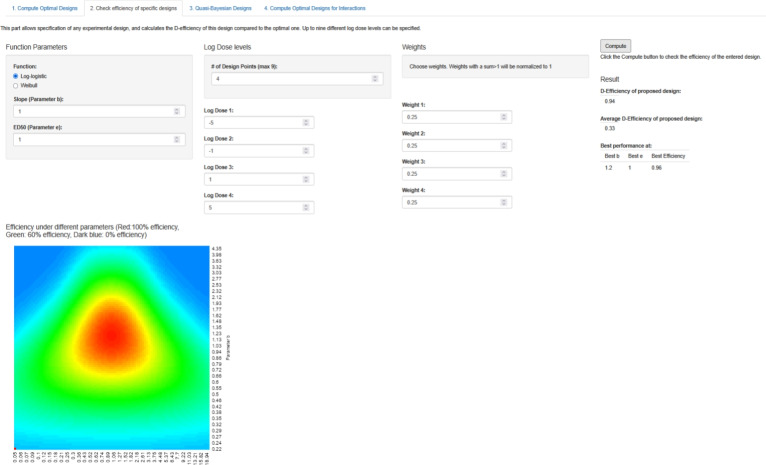
Implementation of the robustness heatmap in DoseResponseDesigns Shiny App

The robustness heatmap has also been implemented into our experimental design Web-App (see Holland-Letz and Kopp-Schneider 2021) available at biostatistics.dkfz.de/DoseResponseDesigns/. On the second tab, up to 9 different dose levels of a design as well as their respective weights can be entered in the “Log Dose Levels” and the “Weights” column, and the App will automatically calculate the efficiency of this design compared to the optimal design, at the assumed parameters specified in the leftmost column, as well as for the most ideal parameter constellation for this design. Furthermore, the App will also calculate the robustness heatmap showing performance at different parameter constellations (see Fig. 3).

## Four robust designs

In the previous section, we have already shown the strictly locally optimal design, which is a design using four distinct dose levels. Here, we will propose several additional designs derived with the aim to be as simple as possible, while maintaining a reasonable performance under different parameter assumptions. We propose three main designs based on the usual setup of equidistant log-steps, and an additional one based on a slightly simplified version of a Bayesian design specifically created to be robust to deviations from the parameter assumptions.

### Equally spaced designs

A very common design includes measurements under a control condition, and then a number of additional dose levels (e.g. eight) with equal weights spaced equally on the log-axis. However, such a design still requires a number of design decisions and thus has potential for optimization. The following decisions are required:

- The number of different dose levels in the experiment. We will consider six, eight and ten dose levels here.
- The step width by which the doses are increased. An increase by a factor of three seems common, and even optimal under some conditions (Holland-Letz and Kopp-Schneider 2015). However, we will still consider a wide range of possible step widths.
- The midpoint around which the other 6/8/10 main dose levels are arranged symmetrically. Usually, this is chosen as a prior guess for the *e*50, and thus requires some prior information. Choosing any other value will decrease general efficiency, and we will thus only consider using the *e*50.
- The weight for the control dose. The control dose might need a larger weight than other doses, as it is the primary setting for evaluation of the control effect parameter *d*.

Often, this class of designs suffers from the lack of a dose level high enough to guarantee the maximum possible effect *c*, which can result in the dose response function not being estimable. To avoid this, we propose always adding the maximum possible dose level to the design, irrespective of the chosen dose grid. Of course, this only applies to experiments where this is technically feasible. Together with the control, such a modified equidistant design will thus have 8/10/12 different dose levels. We will generally assume this additional dose level will have the same weight as the other non-control doses. As a sensitivity analysis, we will also investigate the approach of setting the weight of the maximum dose to a higher value equal to the control weights .

For our optimization, we thus considered (i) 6/8/10 dose levels, (ii) 34 different possible width factors between doses from 0.1 to 3.4 natural log steps in increments of 0.1 log steps. For comparison, the common increase by factors of three corresponds to 1.1 natural log steps between adjacent dose levels. The dose increase by 3.4 log steps is chosen as the maximum step width, as wider spread designs are no longer contained in our assumed design space of practically feasible dose levels ranging from natural log doses of $-10$ to 10. Next, as setting (iii) we set control weights between $10\%$ and $30\%$ in steps of $0.1\%$.

We will optimize in regard to two different optimization metrics, first the performance at the exact initial parameter guess and second the average performance over the whole parameter space defined in Sect. 3.2. As the number of possible parameter combinations is limited here, optimization was done via a simple grid search.

As sensitivity analyses, we will (i) for both metrics also construct an alternative design under the condition that the maximum dose level should have the same weight as the control and (ii) try to adapt the designs to find simpler designs with comparable performance.

### Bayes-optimal design

**Fig. 4:**
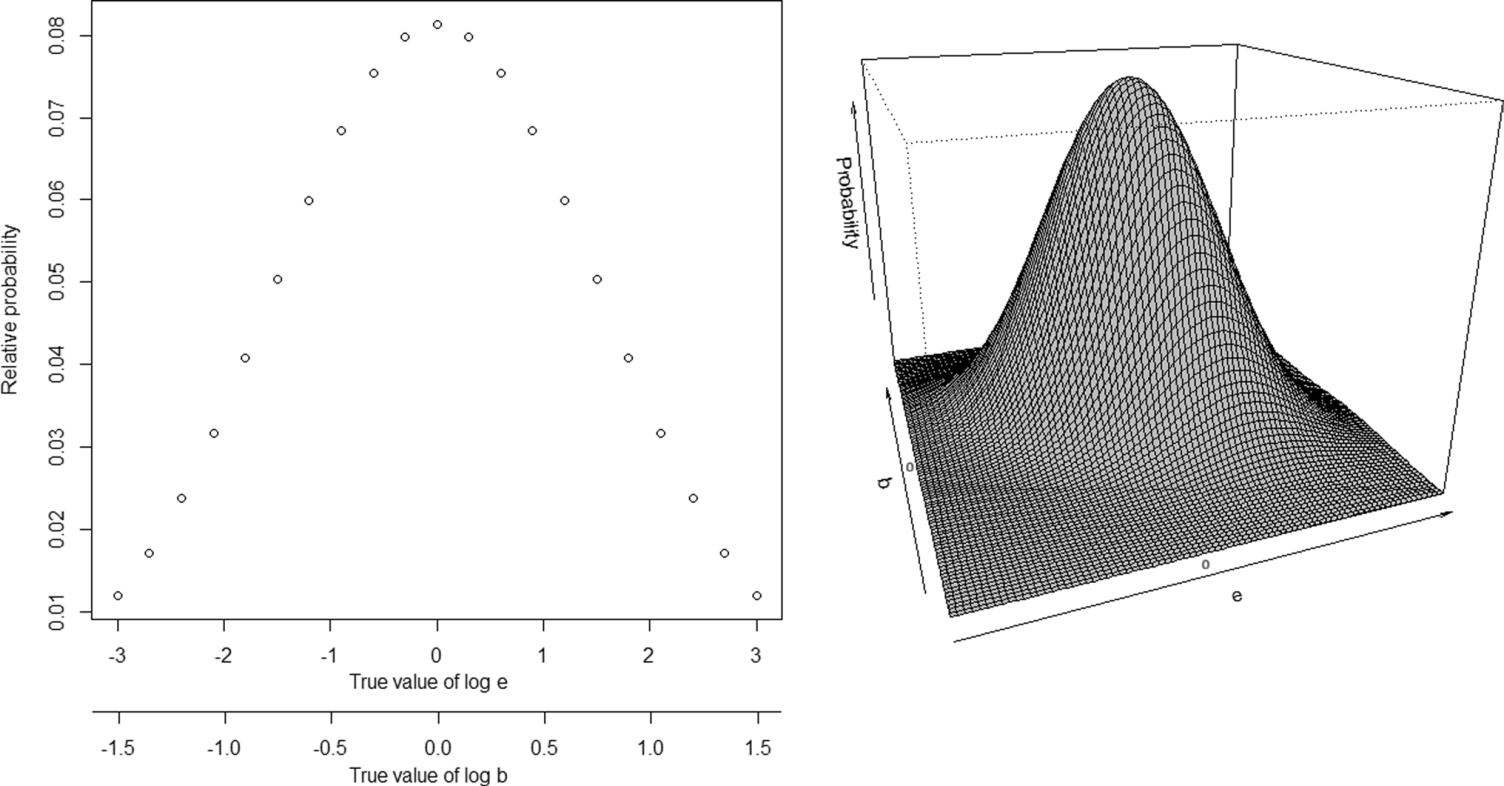
A-priori distribution of parameter values used for Bayes designs. Both parameters follow an independent normal distribution (left), resulting in a bivariate joint distribution of the approximate shape shown on the right

As a final design, we consider a design specifically optimized to perform well under different parameter conditions, with more emphasis placed on parameter sets closer to the center of the range of possible parameters. To do so, a so called (Quasi-)Bayes optimal designs was derived (Chaloner and Verdinelli 1995; Holland-Letz and Kopp-Schneider 2015), which optimizes the performance under a given a-priori distribution of the two relevant parameters. Specifically, a bivariate normal distribution for $\log (b)$ and $\log (e50)$ was selected as an a-priori distribution. Expectation for both log-parameters was assumed as zero, *b* and *e*50 were considered independent and the variances where chosen so that the ends of the specified range for $\log (b)$ and $\log (e50)$ (see Sect. 3.2) fell on the $2.5\%$ and $97.5\%$ Quantiles of the distribution. The resulting marginal probabilities for $\log (b)$ and $\log (e50)$ are illustrated in Fig. 4.

Using the algorithm described in Yu (2010), a design can be found that gives the best *D*-performance on average over this distribution.

### Resulting designs

The main designs resulting from our optimization procedure can be found in Table 1. In each case, the second line in the table for each design specifies the dose levels to be used in the experiment, while the third line specifies which proportion of the observations should be treated with this dose. E.g. if 100 observations are available in total, $10.7\%$ are proposed for a dose level, then 11 observations using this dose level should be performed. Some rounding is often necessary, but generally has a small effect on efficiencies (Pukelsheim and Rieder 1992).

**Table 1:** Different proposed designs and their efficiencies (i) Eflocal: locally at the prior parameter guess, (ii) Efav: average efficiency (iii) Efnorm: Bayesian weighted efficiency with normal weights

Local optimal	Dose Nr	1	2	3	4	5	6	7	8	9	10	Eflocal	Efav	Efnorm
	LogDose	ctrl	−1.04	1.04	max							1.00	0.469	0.604
	Weight	25%	25%	25%	25%									
Equally-spaced I	Dose Nr	1	2	3	4	5	6	7	8	9	10	Eflocal	Efav	Efnorm
	LogDose	ctrl	−1.75	−1.05	−0.35	0.35	1.05	1.75	max			0.897	0.598	0.846
	Weight	25%	10.7%	10.7%	10.7%	10.7%	10.7%	10.7%	10.7%					
Equally-spaced II	Dose Nr	1	2	3	4	5	6	7	8	9	10	Eflocal	Efav	Efnorm
	LogDose	ctrl	−3.5	−2.5	−1.5	−0.5	0.5	1.5	2.5	3.5	max	0.843	0.706	0.949
	Weight	19%	9.0%	9.0%	9.0%	9.0%	9.0%	9.0%	9.0%	9.0%	9.0%			
Equally-spaced III	Dose Nr	1	2	3	4	5	6	7	8	9	10	Eflocal	Efav	Efnorm
	LogDose	ctrl	−3.5	−2.5	−1.5	−0.5	0.5	1.5	2.5	3.5	max	0.867	0.732	0.979
	Weight	19.6%	7.6%	7.6%	7.6%	7.6%	7.6%	7.6%	7.6%	7.6%	19.6%			
Approx. Bayes	Dose Nr	1	2	3	4	5	6	7	8			Eflocal	Efav	Efnorm
	LogDose	ctrl	−3.3	−2.6	−1.9	−1.4	−1.0	−0.6	−0.2			0.910	0.732	0.996
	Weight	18.5%	4.5%	4.5%	4.5%	4.5%	4.5%	4.5%	4.5%					
	Dose Nr	9	10	11	12	13	14	15	16					
	LogDose	0.2	0.6	1.0	1.4	1.9	2.6	3.3	max					
	Weight	4.5%	4.5%	4.5%	4.5%	4.5%	4.5%	4.5%	18.5%					

#### Best local equally spaced design

The optimum performance under the assumed parameter values was achieved with a $6+2$ points design with a step length of 0.7 log steps. We call this design the *Equally spaced I* design (see Table 1).

#### Best average equally spaced design

The optimum average performance over the whole grid of possible parameter settings was achieved identically by an $8+2$ point design with a step length of either 0.9 or 1 log steps, and we propose a step length of 1.0 for simplicity (see Table 1). We call this design *Equally spaced II* design.

#### Best equally spaced designs with strong positive control

Optimizing for local performance under the additional condition that the weights for control and maximum dose should be equal results in a design with $6+2$ dose levels similar to the *Equally spaced I* design, however with a step length of 0.6 log dose levels and weights for the control and max dose of 0.242 each. The performance of this design is better in regard to local efficiency but slightly worse in regard to average and Bayes efficiency (design not shown in Table 1).

Optimizing for average performance results in a design with $10+2$ dose levels and step lengths of 0.8 log steps, yielding an average efficiency of 0.738, while being the largest design so far (design not shown in Table 1). However, by incorporating the feature of equal weights for control and max dose into our *Equally spaced II* design and re-optimizing the weights, we obtain the design labeled *Equally-spaced III* in Table 1, with a comparable performance of 0.732. As this design only has $8+2$ dose levels at a more straightforward step length of 1, while delivering only negligibly worse results, we propose to use this design as a suitable compromise between optimizing average efficiency and simplicity in the context of equally spaced designs.

#### Best Bayesian design

The resulting design was slightly rounded to obtain mostly equal dose weights and can also be found in Table 1. A larger number of 16 different dose levels is required for this design. Note that for the sake of readability, this design is split into two rows in the table.

### Comparison of designs

**Fig. 5:**
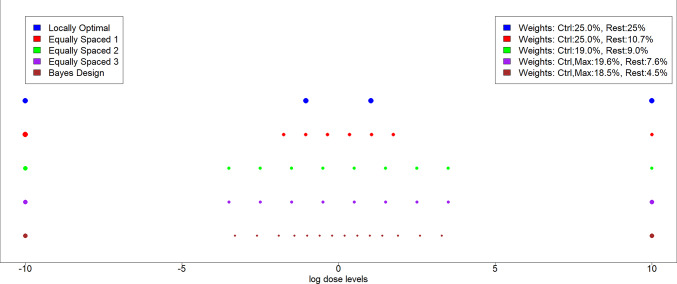
Comparison of proposed dose levels of the locally optimal and the four new presented designs. Point sizes indicate weights. Note that the log dose levels of $-10$ and 10 should in practice be replaced by measurements under control and under the maximum feasible dose, respectively. If the maximum feasible dose falls within the range of the other doses, the generic designs perform poorly and custom designs are required

**Fig. 6:**
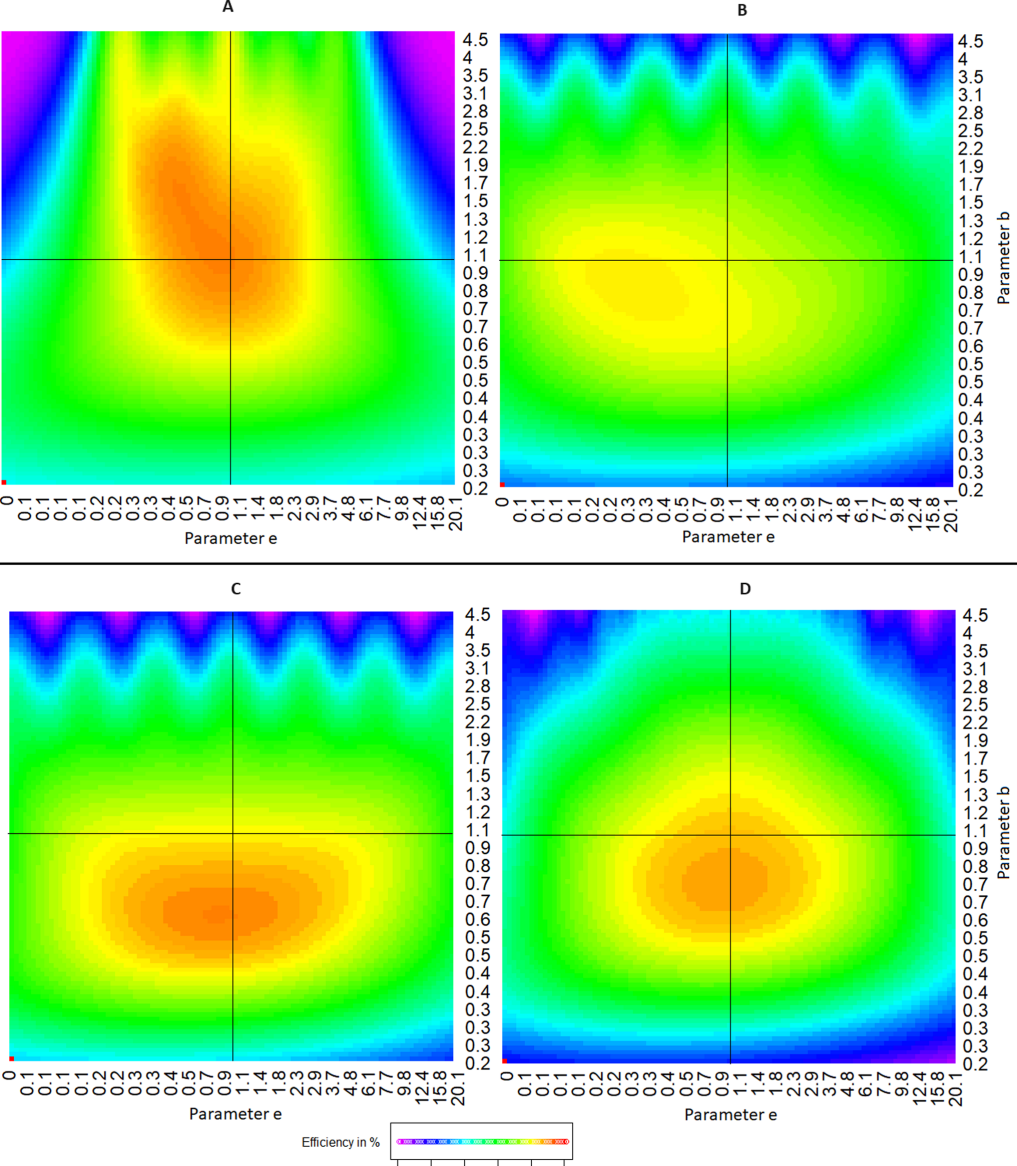
Robustness Heatmaps of the four new presented designs. **A** Equally spaced design I; **B** Equally spaced design II; **C** Equally spaced design III; **D** Bayes optimal design

All four proposed designs are shown in Table 1 and graphically in Fig. 5. The corresponding robustness heatmaps are shown in Fig. 6.

Comparing all four designs in Table 1, we observe that the only design proposing only $6+2$ dose levels is the equally spaced design optimizing local efficiency (Equally spaced design I), while the more robust criterion of average efficiency seems to require $8+2$ dose levels (Equally spaced design II+III). The added complexity of the Bayesian design with $14+2$ doses allows it to perform very well in all three criteria defined in Sect. 3.2 (Local, Average and Bayesian normal efficiency), but at the cost of increased practical effort. However, all of the equally spaced variants still perform decently, and show much better robustness performance compared to the locally optimal 4-point design. Especially the *Equally space III* design comes quite close in performance to the Bayesian design. Comparing the equally spaced designs and the Bayesian design, we notice that the *Equally space III* design and the Bayesian design put similar, larger weights on the max dose, equal to the weights of the control, further supporting the case for the *Equally space III* design. Observing the corresponding heatmaps, different areas of best performance of the designs can be observed. Consistently, all designs still show weakness in situations where *b* is large, which represent extremely steep curves.

### Adaption of standard designs to different conditions

The designs proposed here are designed to be robust within a certain range of different true values of the parameters. As seen in Fig. 4, this range amounts to about 6 log steps for *e*50, and 3 log steps for *b*. For *b*, this range, centered around a value of $b=1$, in our experience covers most *b* values likely to be observed in a cell culture experiment. However, for *e*50 no such general approximation can be made, and any practical experiment therefore still has to be based on a prior guess of this parameter (though the designs will be reasonably robust if this guess is erroneous by no more than a single order of magnitude). Any design should then be centered around this value, instead of the value of 1 used in our proposed designs. Knowledge about *b* is generally harder to obtain in practice, and using the default value of 1 seems to cause little problems in this case. However, if there is prior information available, this should of course be incorporated into the designs. In both cases, the design points $\textit{logdose}_{old}$ given in Table 1 should be shifted as shown in Fig. 1, that is $\textit{logdose}_{new}=ln(e50)+\textit{logdose}_{old}/b$ for all proposed doses in the design. Table 2 shows this exemplary for the locally optimal design and the *Equally Space III* design, but equivalent shifts can be performed for any given design.

**Table 2:** Generic dose levels based on given values of *e*50 and *b*

Locally optimal	Dose Nr	1	2	3	4	5
	LogDose	ctrl	ln(e50) − 1.04/*b*	ln(e50) + 1.04/*b*	max	
	Weight	25%	25%	25%	25%	
Equally-spaced III	Dose Nr	1	2	3	4	5
	LogDose	ctrl	ln(e50) − 3.5/*b*	ln(e50) − 2.5/*b*	ln(e50) − 1.5/*b*	ln(e50) − 0.5/*b*
	Weight	19.6%	7.6%	7.6%	7.6%	7.6%
	Dose Nr	6	7	8	9	10
	LogDose	ln(e50) + 0.5/*b*	ln(e50) + 1.5/*b*	ln(e50) + 2.5/*b*	ln(e50) + 3.5/*b*	max
	Weight	7.6%	7.6%	7.6%	7.6%	19.6%

### A note on well-plates and rounding

All the designs here propose very specific weights (i.e. proportions) to be used. In practice, of course, the proportions are limited by the experimental setup. For example, if the experiment is performed on a single 96-well plate, every well plate represents $1.04\%$ of observations, and finer gradings are not possible. However, in this situation the required weights can be rounded to the nearest feasible value, and the resulting loss of efficiency is generally negligible. For our proposed *Equally space III* designs, the required weights are $19.6\%$ and $7.6\%$. The closest approximation on a 96 well plate would be 19 respectively 7 wells, corresponding to $19.8\%$ and $7.3\%$ of observations and leaving 2 wells empty for now. We propose adding these final wells to the two central log dose levels of $-0.5$ and 0.5, resulting in a final distribution of well plates of (19, 7, 7, 7, 8, 8, 7, 7, 7, 19) when applying our *Equally space III* design from Table 2 on a 96 well plate. The average efficiency of this design is 0.729, that is, $0.3\%$ less than the non-rounded design. Distributing the two unaccounted wells to any other of the 8 central doses instead will change very little and result in a virtually identical efficiency.

Of course, if multiple well plates or larger well plates are available, a closer approximation is possible. In case of a 384 well plate, for example, the best approximation would use 75 wells for control and max dose, plus 29 wells for each of the other 8 doses, leaving once again two surplus wells to be distributed as desired among the eight central dose levels. In this case, the resulting average efficiency is actually indistinguishable from the non-rounded-design at 0.732.

In case that multiple plates will be used in the experiment, generally the same design should be used for all plates, with the option of distributing the unaccounted wells from above differently on each plate, to better match the overall design weights. However, the plate effect should be accounted for in some way in the analysis. In most cases, it should be sufficient to divide all results on a plate by the mean of the control values, before pooling all well results for the main analysis. This accounts for a simple shift in general results from plate to plate, and works well with our proposed designs which all include a decent proportion of control measurements. However, if more sophisticated plate effects are suspected, separate models could be fit for each plate, and the parameters (or other derived measures) could simply be averaged from all plate values for an overall result. Note however, that for log-based measures like the parameters *b* and *e*50, the geometric mean should be used for this step (arithmetic means are usually fine for *c* and *d*).

### A note on sample sizes

Unfortunately, it is not possible to give firm general sample size recommendations for the total numbers of observations to be used in these types of experiments, as these depend directly on the residual error variance in the specific situation (i.e. the expected square distance between the theoretical dose response curve and the actual observed individual observations). However, it seems prudent to choose a sample size that allows actually implementing the chosen design. If, for example, after rounding, a weight of $5\%$ and $13\%$ both results in a single observation taken at the corresponding dose level, little benefit will be achieved from the design. Apart from this, at least in regard to parameter estimation, there is no firm intrinsic requirement to always guarantee at least duplicates or triplicates at every dose level. If the proposed design weights can be reasonably implemented with some doses used only for a single observation, that will still be an effective design. Further observations will simply reduce the variance of all parameter estimates proportionally. However, to retain some flexibility, and robustness against singular failures in observations, making sure that every design dose has at least duplicate or triplicate observations still seems to be advisable. In regard to our Equally spaced III design, requiring duplicates would, after rounding, work out to 28 total observations needed, 2 each for the 8 central doses, plus 6 each for the minimum and maximum doses ($2*8+6*2=28$).

## Summary and conclusion

In this paper, we introduced a graphical depiction of robustness properties of experimental designs, and used the underlying ideas to derive three different equally-spaced designs as well as a robust Bayesian design. All equally spaced designs were shown to be reasonably robust to errors in the prior guess of the parameters, while proving to be more versatile than the locally optimal design, and much simpler than the otherwise superior Bayesian design. Remarkably, due to the inclusion of an additional dose level at maximum available dose, already a standard setup equally spaced design with $6+2$ distinct dose levels (“Equally-spaced I” design) could be shown to achieve almost $90\%$ local efficiency.

Our recommendation is thus to use our “Equally-spaced III” design, which proposes measurements at control, max dose level and in addition at 8 further dose levels spaced 1 natural log level around the expected *e*50 level. Compared to the previous design, the “Equally-spaced III” design provides increased robustness at the cost of two additional dose steps. As this design is still quite simple, few practical obstacles should prevent its use. Furthermore, as most reasonable uncertainties in regard to *b* are covered by our robustness margins, the design requires only an a-priori guess of the *e*50 parameter. If resources are available for a substantially increased number of dose levels, however, the Bayesian design is an even better choice. If a user prefers specific alternative designs, the performance and robustness of these designs can still be judged and visualized using our Shiny-App.

Of course, all our results are based on a specific definition of robustness towards uncertainty in regard to the prior parameter assumptions. We here define reasonable deviations as misspecification within either 3 log-steps for the slope or 6 log-steps for the *e*50-parameter. If even more uncertainty is present, wider spread designs might be needed. However, in such a situation, a two-step approach with a preceeding pilot trial to establish a reasonable a-priori estimate for the parameters might be preferable.

Another limitation of our models is the focus on log-logistic dose response models. While this class of models includes many popular modelling approaches (e.g. hill models, emax models, median effects equations) as special cases, it cannot represent every possible dose response relationship. Our designs should still perform reasonably well for related models such as the log normal model or the asymmetric Weibull model, but in case of severely non-standard behaviour, aspecially non-monotonous dose response relationships, both the usual design and analysis procedures will break down, and a more fundamental investigation of the relationship will be needed.

## Figures and Tables

**Fig. 7:**
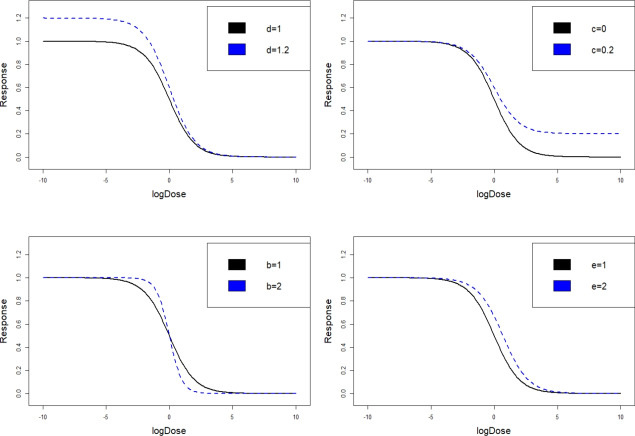
Comparison of log-logistic functions resulting from different values of the four parameters c (lower limit), d (upper limit), b (slope), and e (midpoint). Parameters not defined in the legend follow the standard parametrization $c=0, d=1, b=1, e=1$

## Data Availability

The article is completely theoretical, no data is used, included or available.

## Appendix

### Criteria for evaluation of designs

All criteria are based on efficiency compared to the optimal design $\xi _{opt}$ under the given levels of *b* and *e*50, which of course depends on the parameters *b* and *e*50:

$$\text {Eff}(\xi ,\boldsymbol{\theta })=\text {Eff}(\xi ,(b,e50))=\root k \of {\frac{|M(\xi , \boldsymbol{\theta })|}{|M(\xi _{opt},\boldsymbol{\theta })|}}$$

The three criteria are as follows:

1. The local efficiency measured at an assumed parameter value (b_*prior*_, e50_*prior*_): Eflocal = $\text {Eff}(\xi ,(b_{prior},e50_{prior}))$
2. The average efficiency calculated over every parameter combination (b_1_,…, b_101_) and (e50_1_,…, e50_101_) from the Robustness Heatmap: Efav = $$\frac{1}{10201} \sum _{i=1}^{101} \sum _{j=1}^{101} \text {Eff}$$$$(\xi ,(b_i,e50_j))$$ Note that this criterion corresponds to Bayesian efficiency under the assumption of a uniform a-priori distribution.
3. The Bayesian efficiency calculated over every parameter combination from the Robustness Heatmap, weighted according to the normal distribution based probability densities shown in Fig. 4, denoting the density for *b* as $g(b_i), i \in 1,...,101$ and the density for *e*50 as $h(e50_i), i \in 1,...,101$: $\text {Efnorm}=\sum _{i=1}^{101} \sum _{j=1}^{101} \text {Eff}(\xi ,(b_i,e50_j)) g(b_i) h(e50_j)$

### Log-logistic functions under different parameters

The following Fig. 7 shows the effect of changes in a single parameter on the shape of a log-logistic dose response curve.

### Examples for information matrices

In Tables 3 and 4 we show example information matrices listing the information gained from a single observation under log doses of $-10$ and 0, respectively, in a model under standard parametrization. Diagonal elements indicate information about the four parameters, while non-diagonal element show information about interrelations between parameters, which can also be used for estimation. We observe that an observation at a log dose of $-10$ carries information exclusively about the parameter *d*, while a measurement under a log dose of 0 (i.e. at the *ED*50) carries information about all parameters except the slope *b*. Different log doses will of course carry different information.

In an experiment with multiple observations, these individual information matrices are simply stacked atop each other, that is, added together, to obtain the total information *M* available from the experiment.

**Table 3:** Information matrix for a single observation at log dose $-10$

	c	d	b	e
c	0.00	0.00	0.00	0.00
d	0.00	1.00	0.00	0.00
b	0.00	0.00	0.00	0.00
e	0.00	0.00	0.00	0.00

**Table 4:** Information matrix for a single observation at log dose 0

	c	d	b	e
c	0.25	0.25	0.00	0.12
d	0.25	0.25	0.00	0.12
b	0.00	0.00	0.00	0.00
e	0.12	0.12	0.00	0.06

### Main proposition for generic dose response functions

The following proposition shows conditions which allow designs to be transferred into a shifted design space without change in efficiency. It is based on two ideas:

1. First, we extract from the dose response function only the part (called *h* in the proposition) where dose level and parameters are combined in a nonlinear way.
2. Next, we undo the effect of the parameters by shifting the space of dose levels by the inverse of this combined part (creating shifted dose levels $\tilde{x}$).

If after this shift no nonlinear effect of the parameters on the estimation variances (i.e. the derivatives) remains, the designs on the shifted and non-shifted space of dose levels will be equivalent. More formally, these considerations can be expressed as follows:

#### Proposition

Let $$f:\mathcal{X}\times \varTheta \rightarrow \mathbb {R}$$ be a differentiable function on a design space $$\mathcal{X}=(x_1,...,x_l)$$ of available dose levels, whose parameters $$\boldsymbol{\theta }\in \varTheta \subset \mathbb {R}^k$$ can be separated into $$\boldsymbol{\theta }= ({\boldsymbol{\alpha }},{\boldsymbol{\beta }})$$ with subvectors $${\boldsymbol{\alpha }}=(\alpha _1,\alpha _2)$$ and $${\boldsymbol{\beta }}=(\beta _1,...,\beta _{k-2})$$ so that the function *f* can be split into two scalar subfunctions *g* and *h* with $$f(x,{\boldsymbol{\theta }})$$$$=\alpha _1+\alpha _2 g(h(x,\boldsymbol{\beta }))$$.

Furthermore, assume the subfunction *h* is i) also differentiable and invertible and ii) there exists an additional set of functions $s_i(\boldsymbol{\beta })$ and $t_i(x)$, $i \in (1,...,k-2)$ so that *h* holds $\frac{\partial h(\tilde{x},\boldsymbol{\beta })}{\partial \beta _i} = s_i(\boldsymbol{\beta })t_i(x)$ with $\tilde{x}=h^{-1}(x,\boldsymbol{\beta })$.

Now, let a “parameter shifted design space” for a parameter vector $$\boldsymbol{\theta }= ({\boldsymbol{\alpha }},{\boldsymbol{\beta }})$$ be defined as $$\tilde{\mathcal{X}}$$$$=(\tilde{x}_1,...,\tilde{x}_l)$$$$=(h^{-1}(x_1,\boldsymbol{\beta })$$,…,$$h^{-1}(x_l,\boldsymbol{\beta }))$$. Let $$\xi _A$$ and $$\xi _B$$ be designs on the shifted design spaces $$\tilde{\mathcal{X}}_A$$ and $$\tilde{\mathcal{X}}_B$$ associated with two parameter vectors $$\boldsymbol{\theta }_A$$ and $$\boldsymbol{\theta }_B$$, respectively, with identical weights $${\boldsymbol{w}}={\boldsymbol{w}}_A={\boldsymbol{w}}_B=(w_1,...,w_{l})$$.

Then the design $\xi _B$ is optimal on the design space $\tilde{\mathcal{X}}_B$ under ${\boldsymbol{\theta }}={\boldsymbol{\theta }}_B$ if and only if $\xi _A$ is optimal on the design space $\tilde{\mathcal{X}}_A$ under ${\boldsymbol{\theta }}={\boldsymbol{\theta }}_A$.

#### Proof of Proposition:

Conducting the proof requires a formal definition of the *D*-criterion. Following (Silvey 1980), the *D*-criterion for a design based on *n* observations is given by $|M(\xi , \boldsymbol{\theta })|$ which in turn is proportional to the term $|F_{\boldsymbol{\theta }}F_{\boldsymbol{\theta }}^T|$, with $F_{\boldsymbol{\theta }}=F(\xi , \boldsymbol{\theta }, n)$ given by

2 $$\begin{aligned} F_{\boldsymbol{\theta }}= \left( \begin{array}{ccc} \frac{\partial f(x_1, \boldsymbol{\theta })}{\partial \alpha _1}& ...& \frac{\partial f(x_n, \boldsymbol{\theta })}{\partial \alpha _1} \\ \frac{\partial f(x_1, \boldsymbol{\theta })}{\partial \alpha _2}& ...& \frac{\partial f(x_n, \boldsymbol{\theta })}{\partial \alpha _2} \\ \frac{\partial f(x_1, \boldsymbol{\theta })}{\partial \beta _1}& ...& \frac{\partial f(x_n, \boldsymbol{\theta })}{\partial \beta _1} \\ ...& & \\ \frac{\partial f(x_1, \boldsymbol{\theta })}{\partial \beta _{k-2}}& ...& \frac{\partial f(x_n, \boldsymbol{\theta })}{\partial \beta _{k-2}} \end{array}, \right) \end{aligned}$$

where *n* represents the total sample size to be used in the experiment. The proportionality factor between $|M(\xi , \boldsymbol{\theta })|$ and $|F_{\boldsymbol{\theta }}F_{\boldsymbol{\theta }}^T|$ arises from *n* and the assumed residual error variance $\sigma ^2$ and is not relevant for design purposes.

The proof now relies on the fact that changes in parameters affect the derivatives of functions meeting the conditions given in the Proposition only in two aspects: (i) a multiplicative factor and (ii) a nonlinear factor which is compensated through a shift in the design space.

For the function reparametrization defined in the proposition, the derivatives for the unshifted (${\mathcal {X}}$) and a parameter shifted ($\tilde{{\mathcal {X}}_{\boldsymbol{\theta }}}$) design space look as follows, all based on parameters $\boldsymbol{\theta }=(\boldsymbol{\alpha },\boldsymbol{\beta })$.

Unshifted:

3 $$\begin{aligned} \begin{array}{cclcl} \frac{\partial f(x, \boldsymbol{\theta })}{\partial \boldsymbol{\alpha }}& =& (1,g(h(x,\boldsymbol{\beta }))),\\ \frac{\partial f(x, \boldsymbol{\theta })}{\partial \beta _i}& =& \alpha _2 {g^\prime}(h(x,\boldsymbol{\beta })) \frac{\partial h(x)}{\partial \beta _i},& & i\in (1,...,k-2) \end{array} \end{aligned}$$

Shifted:

4 $$\begin{aligned} \frac{{\partial f(\tilde{x},\boldsymbol{\theta })}}{{\partial \alpha }} =\; & (1,g(h(h^{{ - 1}} (x,\boldsymbol{\beta }),\boldsymbol{\beta }))) \\ =\; & (1,g(x)),i \in (1,...,k - 2) \\ \frac{{\partial f(\tilde{x},\boldsymbol{\theta })}}{{\partial \beta _{i} }} =\; & \alpha _{2} g^{\prime}(h(h^{{ - 1}} (x,\boldsymbol{\beta }),\boldsymbol{\beta }))s_{i} (\boldsymbol{\beta })t_{i} (x) \\ =\; & \alpha _{2} s_{i} (\boldsymbol{\beta })g^{\prime}(x)t_{i} (x) \\ \end{aligned}$$

We observe that the derivatives applied to the new shifted design space are affected by changes in the parameters either not at all (derivatives in direction $\boldsymbol{\alpha }$) or only through the multiplicative term $\alpha _2 s_i(\boldsymbol{\beta })$ (derivatives in direction of $\beta _i, \hspace{0.2cm} i\in (1,..., k-2)$).

We can now investigate the effect of this observation on the actual *D*-criterion. From basic linear algebra we know that multiplicative factors to one row or column in a determinant multiply the full determinant by exactly this factor. Thus, if an identical design $$\xi$$ is used first in a $$\boldsymbol{\theta }_A$$ context, and then in a $$\boldsymbol{\theta }_B$$ context, the criterion value will change as follows: $$\left| {F_{{\user2{\theta }_{B} }} F_{{\user2{\theta }_{B} }}^{T} } \right| = \left| {F_{{\user2{\theta }_{B} }} } \right|^{2} = \left( {\prod\nolimits_{{i = 1}}^{{k - 2}} {\frac{{\alpha _{{2B}} s_{i} (\user2{\beta }_{B} )}}{{\alpha _{{2A}} s_{i} (\user2{\beta }_{A} )}}\left| {F_{{\user2{\theta }_{A} }} } \right|} } \right)^{2}$$$$= \left( {\prod\nolimits_{{i = 1}}^{{k - 2}} {\frac{{\alpha _{{2B}} s_{i} (\boldsymbol{\beta }_{B} )}}{{\alpha _{{2A}} s_{i} (\boldsymbol{\beta }_{A} )}}} } \right)^{2} \left| {F_{{\boldsymbol{\theta }_{A} }} F_{{\boldsymbol{\theta }_{A} }}^{T} } \right|$$. We notice that the multiplicative part in front of the determinant does not depend on the design $$\xi$$.

Assume we have two competing designs $$\xi _1$$ and $$\xi _2$$. Lets say their relative efficiency under $$\boldsymbol{\theta }_A$$ is $$\text {Eff}(\xi _1,\xi _2,\boldsymbol{\theta }_A)$$$$=\root k \of {\frac{|M(\xi _2, \boldsymbol{\theta }_A)|}{|M(\xi _1,\boldsymbol{\theta }_A)|}}=\root k \of {\frac{|F(\xi _2, \boldsymbol{\theta }_A, n)|^2}{|F(\xi _1, \boldsymbol{\theta }_A, n)|^2}}=v$$. Then their efficiency under $$\boldsymbol{\theta }_B$$ is given by

$${\mathrm{Eff}}(\xi _{1} ,\xi _{2} ,\boldsymbol{\theta }_{B} ) = \sqrt[k]{{\frac{{\left( {\prod\nolimits_{{i = 1}}^{{k - 2}} {\frac{{\alpha _{{2B}} s_{i} (\mathbf{\beta }_{B} )}}{{\alpha _{{2A}} s_{i} (\mathbf{\beta }_{A} )}}} } \right)^{2} }}{{\left( {\prod\nolimits_{{i = 1}}^{{k - 2}} {\frac{{\alpha _{{2B}} s_{i} (\mathbf{\beta }_{B} )}}{{\alpha _{{2A}} s_{i} (\mathbf{\beta }_{A} )}}} } \right)^{2} }}\frac{{\left| {F(\xi _{2} ,{\text{ }}\mathbf{\theta }_{A} ,n)} \right|^{2} }}{{\left| {F(\xi _{1} ,{\text{ }}\mathbf{\theta }_{A} ,n)} \right|^{2} }}}}$$$$= \sqrt[k]{{\frac{{\left| {F(\xi _{2} ,\boldsymbol{\theta }_{A} ,n)} \right|^{2} }}{{\left| {F(\xi _{1} ,\boldsymbol{\theta }_{A} ,n)} \right|^{2} }}}}$$$$= \sqrt[k]{{\frac{{\left| {M(\xi _{2} ,{\mathrm{ }}\boldsymbol{\theta }_{A} )} \right|}}{{\left| {M(\xi _{1} ,\boldsymbol{\theta }_{A} )} \right|}}}} = v,$$ the same as before. Thus, knowledge of an optimal design on any shifted design space is equivalent to knowledge of optimal designs on all other parameter shifted spaces. Furthermore, the relative efficiency of any shifted design on a shifted design space will always be the same as the relative efficiency of the original design on its own design space. $$\square$$

### Application to log-logistic function

The four parameter log-logistic function fulfills the conditions of the Proposition, with $\alpha _1=c$, $\alpha _2=d-c$, $\boldsymbol{\beta }=(b,e)$, $g(x)=\frac{1}{1+e^x}$ and finally

$$h(x,\boldsymbol{\beta })= \beta _1 (\ln (x)-\ln (\beta _2))=\beta _1 \ln (\frac{x}{\beta _2})=\ln [(\frac{x}{\beta _2})^{\beta _1}]$$, resulting in

$$h^{-1}(x,\boldsymbol{\beta }) = \beta _2 \exp (\frac{x}{\beta _1}) =: \tilde{x}$$.

As $$\frac{\partial h(x,\boldsymbol{\beta })}{\partial \boldsymbol{\beta }}=(\ln (\frac{x}{\beta _2}), \frac{\beta _1}{\beta _2})$$ and $$\frac{{\partial h(\tilde{x},\boldsymbol{\beta })}}{{\partial {\mathrm{ }}\boldsymbol{\beta }}}$$$$= \left( {\ln \left( {\frac{{\tilde{x}}}{{\beta _{2} }}} \right),\frac{{\beta _{1} }}{{\beta _{2} }}} \right)$$$$= \left( {\frac{x}{{\beta _{1} }},\frac{{\beta _{1} }}{{\beta _{2} }}} \right)$$,

we have $s_1(\boldsymbol{\beta })=\frac{1}{\beta _1}$, $s_2(\boldsymbol{\beta })=\frac{\beta _1}{\beta _2}$, $t_1(x)=x$ and $t_2(x)=1$. As $s_1$ and $s_2$ do depend on the parameters, while $t_1$ and $t_2$ do not, the set of functions fulfills the conditions of the Proposition.

Taking the standard parametrization (0, 1, 1, 1) as $\boldsymbol{\theta }_A$, the shifted design space is just $\tilde{\mathcal{X}}_A=\mathcal{X}$, the original design space. Thus, as the optimal design for the standard parametrization ($\boldsymbol{\theta }_A=(0,1,1,1)$) of a 4-parameter log-logistic function on the standard design space is well known (Holland-Letz and Kopp-Schneider 2015), the proposition allows us to quickly determine optimal designs under many different parameter constellations $\boldsymbol{\theta }_B=({\boldsymbol{\alpha }_B},{\boldsymbol{\beta }_B})=(\alpha _{B1},\alpha _{B2},\beta _{B1},\beta _{B2})$ simply by shifting all (raw) dose levels *x* by $h^{-1}(x,\boldsymbol{\beta }_B)= \beta _{B2} \exp (\frac{x}{\beta _{B1}})$ or, equivalently, all log dose levels to $\frac{\ln (x)}{\beta _{B1}}+ \ln (\beta _{B2})$. Furthermore, the proposition allows us to evaluate arbitrary designs in a wide range of different situations, and both summarize the performance as well as illustrate it in a single graphical plot.
